# High Salt Diet Impacts the Risk of Sarcopenia Associated with Reduction of Skeletal Muscle Performance in the Japanese Population

**DOI:** 10.3390/nu12113474

**Published:** 2020-11-12

**Authors:** Yasuko Yoshida, Keisei Kosaki, Takehito Sugasawa, Masahiro Matsui, Masaki Yoshioka, Kai Aoki, Tomoaki Kuji, Risuke Mizuno, Makoto Kuro-o, Kunihiro Yamagata, Seiji Maeda, Kazuhiro Takekoshi

**Affiliations:** 1 Department of Clinical Laboratory Science, Faculty of Health Sciences, Tsukuba International University, Ibarak 300-0051, Japan; 2 Laboratory of Sports Medicine, Division of Clinical Medicine, Faculty of Medicine, University of Tsukuba, Ibaraki 305-8577, Japan; take0716@krf.biglobe.ne.jp (T.S.); K-takemd@md.tsukuba.ac.jp (K.T.); 3 Faculty of Sport Sciences, Waseda University, Saitama 359-1192, Japan; kosaki.keisei.gm@u.tsukuba.ac.jp; 4 Faculty of Health and Sport Sciences, University of Tsukuba, Ibaraki 305-8577, Japan; maeda.seiji.gn@u.tsukuba.ac.jp; 5 Japan Society for the Promotion of Science, Tokyo 102-0083, Japan; masahiromatsui0708@gmail.com; 6 Graduate School of Comprehensive Human Sciences, University of Tsukuba, Ibaraki 305-8577, Japan; masakiyoshioka1129@gmail.com (M.Y.); fineday0126@gmail.com (K.A.); s1930394@s.tsukuba.ac.jp (T.K.); 7 Faculty of Veterinary Medicine, Okayama University of Science, Ehime 794-8555, Japan; r-mizuno@vet.ous.ac.jp; 8 Division of Anti-aging Medicine, Center for Molecular Medicine, Jichi Medical University, Tochigi 329-0431, Japan; mkuroo@jichi.ac.jp; 9 Department of Nephrology, Faculty of Medicine, University of Tsukuba, Ibaraki 305-8577, Japan; k-yamaga@md.tsukuba.ac.jp

**Keywords:** salt, sarcopenia, renalase, body fat percentage, knee extensor muscle strength, single-leg stance time, maximum gait speed, long seat type body anteflexion, chair rise test

## Abstract

The World Health Organization has recommended 5 g/day as dietary reference intakes for salt. In Japan, the averages for men and women were 11.0 g/day and 9.3 g/day, respectively. Recently, it was reported that amounts of sodium accumulation in skeletal muscles of older people were significantly higher than those in younger people. The purpose of this study was to investigate whether the risk of sarcopenia with decreased muscle mass and strength was related to the amount of salt intake. In addition, we investigated its involvement with renalase. Four groups based on age and salt intake (“younger low-salt,” “younger high-salt,” “older low-salt,” and “older high-salt”) were compared. Stratifying by age category, body fat percentage significantly increased in high-salt groups in both younger and older people. Handgrip strength/body weight and chair rise tests of the older high-salt group showed significant reduction compared to the older low-salt group. However, there was no significant difference in renalase concentrations in plasma. The results suggest that high-salt intake may lead to fat accumulation and muscle weakness associated with sarcopenia. Therefore, efforts to reduce salt intake may prevent sarcopenia.

## 1. Introduction

Japan has a large aging population; the 2019 White Paper on Aging Society reported that 28.1% of the population is over the age of 65 years [1]. In addition, soaring medical costs have become a social problem. One of the causes is that there is a discrepancy between the average life expectancy and the period of healthy life expectancy during which a person can live an independent life [1]. Frailty, the decline in motor and cognitive function in individuals with advanced age, is one of the reasons healthy life expectancy remains lower than average life expectancy. The main cause for frailty is sarcopenia, a progressive age-related weakness of the muscles. The definition of sarcopenia was based on the criteria of the European Working Group on Sarcopenia in Older People (EWGSOP) in 2010 [2]. In Japan, the criteria of the Asian Working Group for Sarcopenia (AWGS) are generally recommended for diagnosis, and in 2017 the Japanese Association on Sarcopenia and Frailty created the sarcopenia clinical practice guidelines [3,4,5].

The main cause of sarcopenia is aging [2,3,4,6]. Secondary factors include physical inactivity, illness, and nutrition, and it is likely that multiple factors overlap to cause sarcopenia. Specifically, with regard to nutrition, reports indicate that deficiency of protein and vitamin D intake increases the risk of sarcopenia [7,8,9,10,11,12,13,14,15,16,17,18,19,20,21]. The leading problem regarding nutrients in Japanese people is high-salt intake. The World Health Organization (WHO) has recommended an intake of no more than 5 g of salt/day [22]. In Japan, there is an average salt intake of 11.0 g/day for men and 9.3 g/day for women. Recently in Japan, salt intake has been declining slightly each year [23].

Many studies report that excessive salt intake is one of the causes of various diseases, such as hypertension [24,25], where sodium in the skeletal muscle accumulates more in older people than in younger people, and patients with refractory hypertension have increased tissue sodium(Na (+)) content when compared with normotensive controls [26]. In addition, the sodium-potassium-chloride symporter 1 (NKCC1) is highly expressed in mammalian skeletal muscle. The physiologic function of NKCC1 in myogenesis is unclear. However, NKCC1 protein levels increased skeletal myoblast differentiation, and NKCC1 inhibitors markedly suppressed skeletal myoblast differentiation [27]. It has also been reported that excess sodium leads to a downregulation of expression of NKCC [28]. Therefore, we hypothesized that the risk of sarcopenia, which is associated with decreased muscle mass and strength, is related to the amount of salt intake, because Na (+) is stored in tissues and NKCC1 is involved in muscle hypertrophy and suppression.

Incidentally, renalase, a recently discovered enzyme released by the kidneys, may break down blood-derived catecholamine and regulate blood pressure. A report of loss of renalase function can result in increased blood pressure (hypertension), increased heart rate (tachycardia), increased vascular resistance (vasoconstriction), and increased catecholamine response [29]. Human and animal studies have suggested that high levels of dietary salt may lower blood and renal renalase levels. Due to blood pressure increasing with salt intake, the renalase levels that breaks down catecholamines, may also be reduced [30,31,32]. For the relationship between renalase and skeletal muscle, exercise load increased blood levels of renalase, independent of renal secretion, and increased gene expression of renalase in skeletal muscles [33,34,35]. In a study based on disuse atrophy, there was a study that showed muscle proteolysis decreased and muscle mass increased in renalase knockout mice [36]. On the other hand, skeletal muscle has β2 adrenergic receptors involved in catecholamines, and β2-adrenergic receptor stimulation increases muscle mass by promoting muscle protein synthesis and/or attenuating protein degradation. However, excessive stimulation of -adrenergic receptors negates their beneficial effects [37]. By these reports, we considered that renalase, which is related to catecholamines that increase with salt intake, may also be involved in skeletal muscle atrophy. Therefore, we speculated that the decreased level of renalase might be a marker for sarcopenia.

We aimed to clarify the relationship between salt intake, blood renalase concentrations, and sarcopenia risk for Japanese adults.

## 2. Materials and Methods

### 2.1. Study Design

Healthy adult volunteers (*n* = 122, age (standard deviation [SD]): 56.3 (12.3) years) were recruited from the community using local advertisements, and participants were selected from Tsukuba and nearby urban areas (Ibaraki, Japan). Participants with an estimated glomerular filtration rate of 90 mL/min/1.73 m^2^ or less were excluded because renal function is involved in sodium uptake and renalase levels [24,30,31,32,38,39,40]. In addition, eight participants who showed more than four times the standard deviation of the mean blood test of all 122 participants were excluded, because there was a possibility of other diseases. The items are renalase, Interleukin-6 (IL-6), triglyceride (TG), insulin, glucose (Glu), glycosylated hemoglobin (HbA1c), aspartate transaminase (AST), and alanine aminotransferase (ALT). Therefore, 114 participants were analyzed.

We calculated estimated salt intake using spot urine testing and divided the participants into two groups with an average estimated salt intake of 9.37 g/day [1]. The participants with lower salt intake than average was designated as “low-salt,” and the participants with a higher intake were designated as “high-salt.” Furthermore, since the age range of the selection criteria for this study is 20 years and over, volunteers from a very wide range of age groups participated, we also analyzed after stratifying by age. Participants were divided according to the overall average age (56.3 (12.3)) designated “younger” and “older.” That is, “younger” is younger than the average age of participants and “older” is older than the average age. Overall, the participants were divided into four groups (“younger low-salt,” “younger high-salt,” “older low-salt,” “older high-salt”) and blood test results, anthropometric measurements, and assessment of physical performance were compared.

This study was approved by the Ethical Review Board of University of Tsukuba, and all participants provided written, informed consent as per the requirements of the Declaration of Helsinki (IRB approval No. H30-161).

### 2.2. Data Collection

#### 2.2.1. Blood Test and Urinalysis

Participants were asked to avoid strenuous exercise, live a normal life the day before and were banned from eating and drinking except water after 20:00 p.m., before testing to control for exercise and nutrition. They were asked to arrive at the laboratory between 8:00 a.m. and 11:00 a.m.

First, participants provided urine samples. We conducted urinalysis to measure the concentration of sodium and creatinine at Tsukuba i-Laboratory Limited Liability Partnership (Ibaraki, Japan), using the ion-selective electrode method to measure urinary sodium and the enzyme method to measure urinary creatinine. We calculated the 24-h salt intake using the following formula [41,42]:

Estimated 24 h Creatinine (mg/day)  =  −2.04  ×  age  +  14.89 × weight (kg) + 16.14  ×  height (cm) − 2244.45,(1)

Estimated 24 h urinary sodium (mEq/day)  = 21.98  ×  urinary sodium/10/(1) ^0.392^, (2)

Estimated 24-h salt intake (g/day) = (2)/17.(3)

Next, blood samples were collected from the antecubital vein. In this study, we measured some parameters in the blood that could pose a risk for sarcopenia. IL-6 (pg/mL) for inflammation, urea nitrogen (UN) (mg/dL) and cystatin C (CysC) (mg/L) for renal function, TG (mg/dL) for fat accumulation that causes obesity, albumin (Alb) (g/dL) for nutritional status, Glu (mg/dL), insulin (μU/mL) and HbA1c (%), for glucose metabolism, and AST (U/L) and ALT (U/L) for liver function. In addition, since renalase (mg/L) has been reported to be involved in blood pressure control function and skeletal muscle atrophy by metabolizing catecholamines, it was measured as a marker candidate for sarcopenia. Whole blood was used to measure HbA1c, which was measured using the enzyme method. The remaining blood was brought to room temperature of 20 to 25 °C and then centrifuged at 3000 rpm for 10 min to obtain a serum. It was then stored at −80 °C until measurement. Serum was used for measurement of concentrations of the following parameters: IL-6, UN, CysC, TG, Alb, insulin, Glu, AST, ALT, and renalase. The measurement was carried out at Tsukuba i-Laboratory Limited Liability Partnership (Ibaraki, Japan) by chemiluminescent enzyme immunoassay (insulin), ultraviolet (UN, Glu), latex coagulating nephelometry (CysC), the enzyme method (TG), the bromocresol purple method (Alb), and the Japan Society of Clinical Chemistry (JSCC) Standardization Corresponding Method (AST, ALT). IL-6 was measured by Chemiluminescent Enzyme Immunoassay at Jichi Medical University Hospital. Renalase was measured by enzyme-linked immunosorbent assay (ELISA) using the FAD-Dependent Amine Oxidase ELISA Kit (Cloud-Clone Corp, Houston, TX, USA). In addition, the estimated glomerular filtration value (eGFR) was calculated using the renal function presumption formula and serum CysC concentrations to evaluate kidney function. Moreover, eGFR using the serum CysC concentration was calculated using the equation for Japanese people as shown in the chronic kidney disease (CKD) clinical practice guidelines [40] as follows: eGFR (mL/min/1.73 m^2^) = (104 × [Concentration of serum CysC (mg/dL)]^−1.019^ × 0.996^(Age)^ − 8”.

#### 2.2.2. Anthropometric Measurements

Heights were measured using a stadiometer. Weight and body compositions were then measured using bioelectrical impedance analysis (Inbody 770; Inbody, Tokyo, Japan). The body composition measurement included skeletal muscle mass index (SMI; kg/m^2^), body fat percentage (BFP; %), and mass of muscle of upper limbs (ULMM; kg) and lower limbs (LLMM; kg) [43,44]. Then we calculated the body mass index (BMI; kg/m^2^)

#### 2.2.3. Assessment of Physical Performance

Handgrip strength (HGS) (kg), knee extensor muscle strength (KES) (kg), single-leg stance time (SLT) (s), maximum gait speed (MGS) (m/s), long seat type body anteflexion (FleX) (cm), and the chair rise test (30CS) (number of repetitions) were used as indices of physical performance [3,4,5,45,46,47,48,49,50,51]. HGS was determined using a handheld dynamometer (T.K.K.5401; Takei Kiki Kogyo, Niigata, Japan). The participants being tested alternated between their left and right hand and were measured twice. The mean of the maximal values for each hand was used for analysis [49,50]. Then, the HGS was divided by body weight (BW). KES was determined using a handheld dynamometer (μTas F-1; ANIMA, Tokyo, Japan). Both lower limbs were measured twice, and the average of their highest score was registered as the result. Then, the KES was divided by body weight [48,50]. SLT was measured as an indicator of balance performance. Participants balanced as long as possible on one leg with their eyes open. The participant could choose which foot on which to balance. The maximum value was 60 s. If the maximum value was achieved, it was completed once; if not, the test was carried out twice and the longest time was used [47,50]. MGS was calculated using the time to cover a distance of 10 m on a straight walking course. To ensure that the participants reached their maximal walking speed, the 10 m was measured within a 16-m track [46,50]. FleX was determined using the long seat type body anteflexion measurement device (T.K.K.5412; Takei Kiki Kogyo, Niigata, Japan) [45]. The measurement was performed twice, and the largest value was used as the data. Muscular function of lower extremities was measured by 30CS as described by Jones et al. [51]. Participants were instructed to stand up as often as possible within 30 s. The score was the total number of stands executed correctly (stretched knee and hip) within 30 s. The measurement was performed only once.

### 2.3. Statistical Analysis

Data were analyzed using SPSS Statistics 26.0 (IBM, Armonk, IL, USA). Significance was set at *p* < 0.05. All values are expressed as mean (standard deviation) or median [interquartile range] unless stated otherwise. A chi-square test was performed to confirm that there was no difference in the ratio of males and females in each group. Normal distribution was confirmed by a Kolmogorov–Smirnov test. In the comparison between two groups, an independent t-test was performed in the case of normal distribution, and the Mann–Whitney U test was performed in other cases. For unequal variance, Welch’s correction was applied. The correlation was analyzed by Pearson’s correlation coefficient and Spearman’s correlation coefficient. In the comparison between the four groups, one-way analysis of variance (ANOVA) for normality was performed, and Kruskal–Wallis for non - normality testing was performed otherwise. If there was a significant difference, either the Tukey test or the Bonferroni test was applied as a post hoc test. For multiple analysis, multiple linear regression analysis was performed with salt intake as the dependent variable.

## 3. Results

### 3.1. Comparison with Estimated Daily Salt Intake

The normality of each parameter is shown in Table 1. All parameters except BFP, HGS/BW, KES/BW, Flex, UN, Alb, HbA1c, and AST showed a non-normal distribution. The comparison between the “low-salt” and “high-salt” groups is shown in Table 1. There were no significant differences in sex ratio, age, and height. However, there were significant differences in SBP, DBP, weight, BMI, BFP, HGS/BW, Flex, 30CS, IL-6, TG, insulin, and ALT.

### 3.2. Correlation with Estimated Salt Intake

The correlations between estimated salt intake and sarcopenia-related parameters are shown in Table 2. As for the correlation coefficient, the correlation coefficient of Pearson was analyzed in the case of normality, and the correlation coefficient of spearman was analyzed in the case of non-normality. There were significant differences in SBP, Weight, BMI, SMI, ULMM, BFP, HGS/BW, Flex, 30Cs, IL-6, TG, Insulin, and ALT. SBP, Weight, SMI, ULMM, HGS/BW, Flex, 30CS, IL-6, TG, Insulin, and ALT was weakly correlated and BMI and BFP was moderately correlated.

### 3.3. Comparison of Estimated Salt Intake and Age

The comparison between the “younger low-salt,” “younger high-salt,” “older low-salt,” and “older high-salt” groups is shown in Table 3. There were significant differences in SBP, DBP, weight, BMI, SMI, ULMM, LLMM, BFP, HGS/BW, 30CS, IL-6, UN, TG, Glu, HbA1c, and AST.

**Figure 1:**
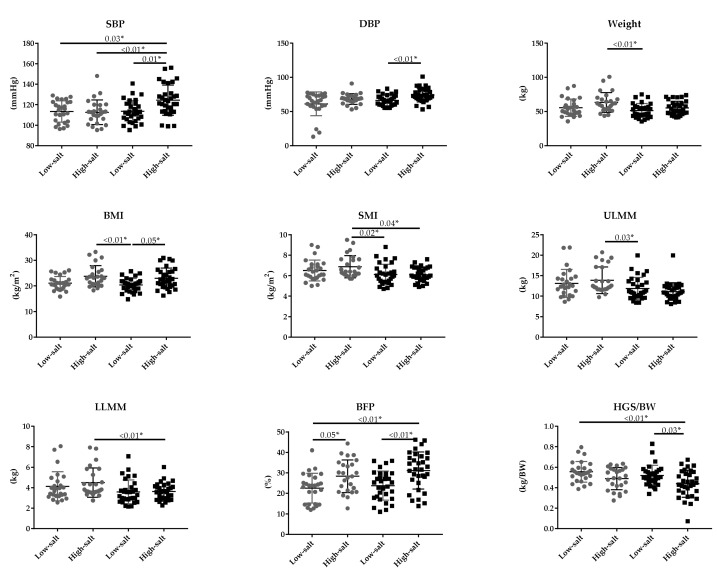

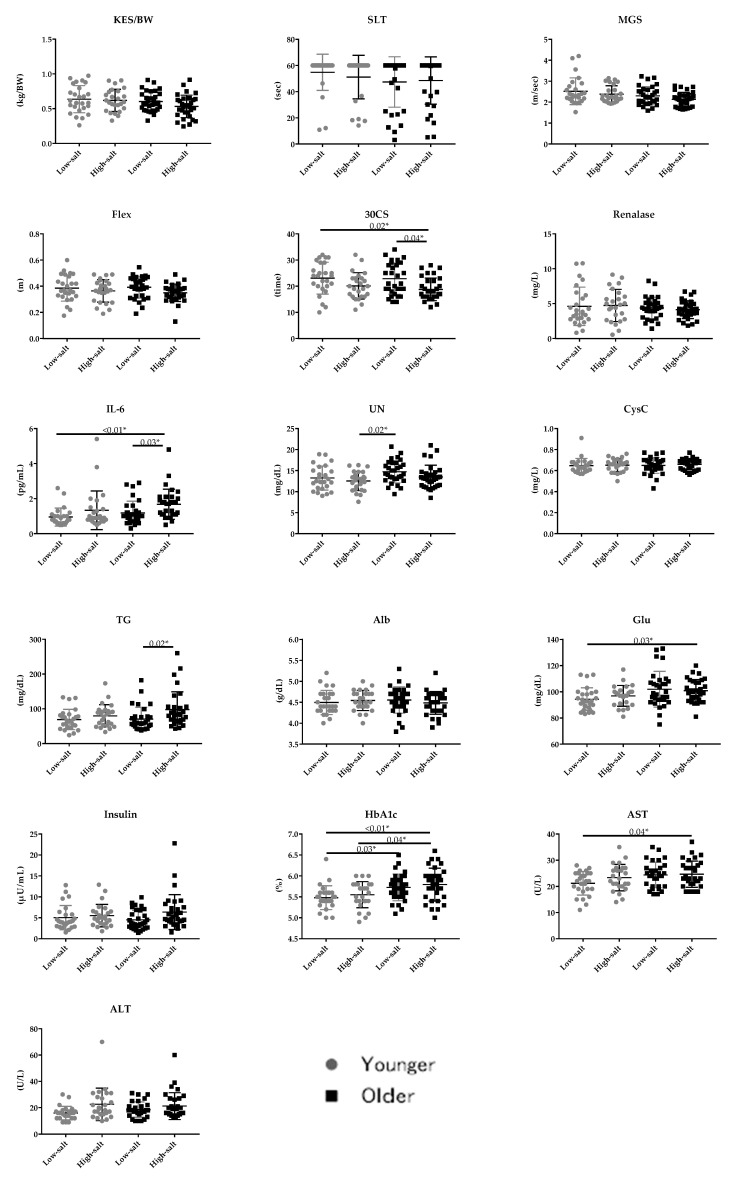
For comparison of the four groups, one-way ANOVA or Kruskal-Wallis test was performed, followed by a post hoc test. *: *p* < 0.05. sec: seconds, BW: body weight, SBP: systolic blood pressure, DBP: diastolic blood pressure, BMI: body mass index, SMI: skeletal muscle mass index, ULMM: mass of muscle of upper limbs, LLMM: mass of muscle of lower limbs, BFP: body fat percentage, HGS/BW: handgrip strength/weight, KES: knee extensor muscle strength, SLT: single-leg stance time, MGS: maximum gait speed, Flex: long seat type body anteflexion, 30CS: chair rise test, IL-6: interleukin-6, UN: urea nitrogen, CysC: cystatin C, TG: triglyceride, Alb: albumin, Glu: glucose, HbA1c: glycosylated hemoglobin, AST: aspartate transaminase, ALT: alanine aminotransferase.

In the comparison between the four groups, one-way analysis of variance (ANOVA) for normality was performed while Kruskal-Wallis was performed for non-normality testing. Significant differences were found in SBP, DBP, Weight, BMI, SMI, ULMM, LLMM, BFP, HGS/BW, 30CS, IL-6, UN, TG, Glu, HbA1c, and AST. A post hoc test was calculated only on the parameters that were significantly different. Figure 1 shows the p-values of the parameters that were significantly different in the post hoc test. When Younger and Older are stratified, significant differences were found in SPB, DBP, BMI, HGS/BW, 30CS, IL-6, and TG only in the older group. BFP was significantly different in both younger and older groups. weight, SMI, ULMM, LLMM, UN, Glu, HbA1c, and AST were significantly different between the younger group and the older group.

### 3.4. Multivariate Analysis of Estimated Salt Intake and Sarcopenia Parameters

Multiple linear regression analysis used salt intake as the dependent variable. The independent variable populated all parameters related to sarcopenia risk. We then implemented the stepwise method. The adopted factors were BFP, ULMM, IL-6, SLT, LLMM, 30CS, ALT, and insulin, and the multiple regression equation for predicting salt intake (Y) was Y = 0.12 × (BFP) + 1.44 × (ULMM) + 0.46 × (IL-6) + 0.03 × (SLT) − 0.42 × (LLMM) − 0.08 × (30CS) + 0.05 × (ALT) − 0.14 × (insulin) + 5.09. The multiple correlation coefficient was 0.46, the adjusted coefficient of determination was 0.42, and the adjusted multiple correlation coefficient was 0.68. The value of the Burbin Watson test was 2.40. Residual analysis showed that the model was reliable.

## 4. Discussion

This study was conducted with a focus on salt intake, which pertains to nutritional intake. It is known that the salt intake of Japanese people is the highest in the world [52,53]. In this study, the estimated daily salt intake using the urine test values was high, with an average value of 9.32 g/day (2.14). This amount is close to the reported average Japanese salt intake and is almost twice the recommended amount given by the WHO [22,23].

All parameters that showed a significant difference are values indicating the degree of obesity and the amount of fat in the body. In addition, salt intake, BFP, and BMI showed positive correlations. These results suggest that excessive salt intake may contribute to fat accumulation and are consistent with previous studies reporting an association between excessive salt intake and obesity [18,54,55,56]. One factor that predicts sarcopenia is the increase of fat in muscle [57,58,59,60]. Excessive salt intake can lead to fat accumulation and sarcopenia risk, however, participants who consume excess salt may not have dietary controls other than salt, which may lead to fat accumulation and obesity. Since no dietary survey was conducted in this study, this cannot be clarified. In terms of assessment of physical performance, some parameters, such as muscle weakness, were significantly lower in the high-salt group than the low-salt group. Muscle weakness is one of the causes of sarcopenia [50,61]. However, according to the criteria of the AWGS [3], there were only five in the older group that had reduced grip strength. The high-salt: low-salt group ratio was 3:2. Furthermore, no decrease in the parameter (SIM) indicating muscle mass loss could be seen. Therefore, in this study, we can only say that there is a possibility of sarcopenia risk, and not sarcopenia. Regarding blood tests, the fact that IL-6 and insulin were significantly higher in the high-salt group may suggest insulin resistance. Insulin resistance contributes to obesity and aging, and skeletal muscle insulin secretion resistance is involved in the pathogenesis of sarcopenia. IL-6 has also been shown to induce insulin resistance. Therefore, when homeostasis model assessment as an index of insulin resistance (HOMA-IR) was calculated [62], the average value of low salt content and high salt content was 1.18 (0.69):1.49 (0.91), and HOMA-IR also increased significantly. Increased ALT in blood tests is associated with liver dysfunction. There are many reports of the risk of sarcopenia due to liver dysfunction, and there are also diagnostic criteria for liver disease in Japan [4]. Increases in all these parameters are associated with sarcopenia risk [4,12,13,14,15,16,17,18,19].

Next, we investigated the relationship between estimated salt intake, age, and a possibility of sarcopenia risk. There were 16 parameters with a significant difference. Parameters were stratified into the younger and older groups, the items that showed a significant difference only in older groups were the parameters related to fat and the parameters indicating the decrease in muscle strength or physical function. In addition, only BFP had significantly higher mean values in the high-salt group compared to the low-salt group in both the younger and older groups. In other words, the results of this study showed that excessive intake of salt was related with accumulated fat parameters in the bodies in both younger and older groups. The comparison of muscle strength in this study did not follow the sarcopenia definition of EWGSOP and AWGS, but HGS/BW, which indicates muscle strength, and 30CS, which indicates physical function, were significantly decreased in the high-salt group [2,3,4]. Therefore, in the older group, the high-salt group had fat accumulation and muscle weakness.

The expression of the renalase may also be related to salt intake and sarcopenia [30,31,32]. However, we observed no significant relation between blood renalase levels and sarcopenia in this study. A previous study reported that the daily intake of salt was 18 g, much higher than the 4.0–13.8 g/day of this experiment [30]. The results of this study did not reveal a link between renalase, salt intake, and sarcopenia.

Our study compared people with high and low salt intakes and found that those with high-salt intake had higher fat-related parameters. However, we could not investigate causal relationships and mechanisms. For example, fat accumulation is associated with a variety of factors. Salt intake can contribute to fat accumulation. It can also be inferred that fat accumulation changes depend on diet and physical activity. It cannot be ruled out that factors that have not been measured or considered may have confounded the observational results in this study. First, a detailed dietary and physical activity survey would be required to elucidate the association with salt intake. A second limitation of this study is water intake. “Inbody 770”, which measures body composition, is affected by the amount of water in the body because it uses the bioelectrical impedance analysis method. Therefore, if the body composition is to be measured strictly, water restriction should be controlled from the previous day. The third, this study recruited adults over the age of 20, participants ranging from 22 to 81 years old, with an average age of 56 years. Further recruitment of participants over the age of 60 or 65 should have been recruited to investigate the association between salt intake and sarcopenia.

Our study does not directly show that excessive salt intake causes sarcopenia, but a diet with excessive salt is associated with fat accumulation and muscle weakness. Furthermore, aging without improving diet can lead to the development of sarcopenia. In addition, past papers have reported that skeletal muscle mass and skeletal muscle strength are maximized in the 20s and 30s [63], and there is a standard that the target age for sarcopenia is 60 or 65 years [3]. On the other hand, there are reports targeting people over 40 years old [64]. Controlling the diet from a young age can prevent various diseases, including the prevention of sarcopenia.

## 5. Conclusions

This study analyzed the relationship between salt intake and the risk of sarcopenia in the Japanese population. As a result, the parameters related to obesity were significantly increased and the parameters related to muscle strength were significantly decreased in the group of high-salt intake compared with the low-salt intake group. In addition, the results were more significant in the older group than the younger group. Excessive salt intake may be associated with risk of sarcopenia, although further analysis is needed.

## Figures and Tables

**Table 1:** Comparison of sarcopenia-related parameters by estimated salt intake.

	Low—Salt	High—Salt	Normality *p* value	T Test
				*p* Value
Sample size (n)	57	57	-	-
Salt intake (g/day)	7.62(1.31)	11.11(1.16)	0.63	<0.01 *
Male/Female (*n*/*n*)	11/46	8/49	-	0.45 ^✝^
Age (year) ^§^	56.00[49.00–63.00]	56.00[50.5–65.00]	<0.01 *	0.60
SBP (mmHg) ^§^	113.00[104.33–122.00]	117.33[110.33–128.00]	<0.01 *	0.05 *
DBP (mmHg) ^§^	65.67[60.00–73.00]	70.33[66.17–76.00]	0.04 *	0.01 *
Height (m) ^§^	1.59 [1.53–1.66]	1.57[1.52–1.61]	<0.01 *	0.28
Weight (kg) ^§^	51.20[45.30–58.30]	57.60[48.75–66.25]	<0.01 *	0.01 *
BMI (kg/m^2^) ^§^	20.61[18.88–21.89]	22.69[20.28–25.58]	<0.01 *	<0.01 *
SMI (kg/m^2^) ^§^	6.10[5.50–6.90]	6.20[5.80–6.85]	<0.01 *	0.34
ULMM (kg) ^§^	3.56[2.95–4.32]	3.67[3.24–4.35]	<0.01 *	0.24
LLMM (kg) ^§^	11.69[10.04–14.19]	11.78[10.44–12.82]	<0.01 *	0.88
BFP (%)	23.21(7.15)	29.91(8.65)	0.10	<0.01 *
HGS/BW (kg/BW)	0.54(0.10)	0.46(0.12)	0.23	<0.01 *
KES/BW (kg/BW)	0.62(0.17)	0.57(0.16)	0.16	0.13
SLT (sec) ^§^	60.00[48.05–60.00]	60.00[38.24–60.00]	<0.01 *	0.72
MGS (m/sec) ^§^	2.28[2.04–2.61]	2.18[1.99–2.44]	<0.01 *	0.14
Flex (m)	0.39(9.08)	0.36(7.54)	0.75	0.04 *
30CS (time) ^§^	24.00[19.00–29.00]	18.00[16.00–23.00]	<0.01 *	<0.01 *
Renalase (mg/L) ^§^	4.26[2.93–5.46]	4.24[3.30–5.38]	<0.01 *	0.90
IL-6 (pg/mL) ^§^	1.00[0.65–1.20]	1.20[0.90–1.90]	<0.01 *	<0.01 *
UN (mg/dL)	14.04(2.81)	13.07(2.61)	0.10	0.06
CysC (mg/L) ^§^	0.65[0.61–0.69]	0.67[0.62–0.70]	<0.01 *	0.27
TG (mg/dL) ^§^	61.00[46.00–78.00]	83.00[59.50–100.50]	<0.01 *	<0.01 *
Alb (g/dL)	4.53(0.30)	4.51(0.26)	0.10	0.77
Glu (mg/dL) ^§^	96.00[91.00–106.00]	99.00[92.50–104.00]	<0.01 *	0.28
Insulin (μU/mL) ^§^	3.90[2.90–6.10]	4.90[3.75–7.15]	<0.01 *	0.02 *
HbA1c (%)	5.61(0.33)	5.69(0.38)	0.11	0.27
AST (U/L)	22.93(4.93)	24.11(5.03)	0.70	0.21
ALT (U/L) ^§^	17.00[14.00–19.00]	18.00[15.00–27.00]	<0.01 *	0.05 *

These data are shown as mean (standard deviation) or median [interquartile range]. For the normality test, the Kolmogorov–-Smirnov test was performed. Two-group comparison is the result of the independent T test or Mann–-Whitney-U test [§]. The statistics of male and female is the chi-square test [✝]. *: *p* < 0.05. sec: seconds, BW: body weight, SBP: systolic blood pressure, DBP: diastolic blood pressure, BMI: body mass index, SMI: skeletal muscle mass index, ULMM: mass of muscle of upper limbs, LLMM: mass of muscle of lower limbs, BFP: body fat percentage, HGS/BW: handgrip strength/weight, KES: knee extensor muscle strength, SLT: single-leg stance time, MGS: maximum gait speed, Flex: long seat type body anteflexion, 30CS: chair rise test, IL-6: Interleukin-6, UN: urea nitrogen, CysC: cystatin C, TG: triglyceride, Alb: albumin, Glu: glucose, HbA1c: Glycosylated hemoglobin, AST: Aspartate transaminase, ALT: Alanine aminotransferase.

**Table 2:** Correlation of sarcopenia-related parameters based on estimated salt intake.

	Correlation Coefficient	
	*p* value	r value
Age (year) ^§^	0.54	-
SBP (mmHg) ^§^	0.03 *	0.21
DBP (mmHg) ^§^	0.07	-
Height (m) ^§^	0.74	-
Weight (kg) ^§^	<0.01 *	0.39
BMI (kg/m^2^) ^§^	<0.01 *	0.49
SMI (kg/m^2^) ^§^	0.03 *	0.21
ULMM (kg) ^§^	0.01 *	0.24
LLMM (kg) ^§^	0.42	
BFP (%)	<0.01 *	0.49
HGS/BW (kg/BW)	<0.01 *	−0.38
KES/BW (kg/BW)	0.14	−
SLT (sec)^§^	0.46	−
MGS (m/sec) ^§^	0.24	−
Flex (m)	0.03 *	−0.20
30CS (time) ^§^	<0.01 *	−0.32
Renalase (mg/L) ^§^	0.67	−
IL-6 (pg/mL) ^§^	<0.01 *	0.31
UN (mg/dL)	0.25	−
CysC (mg/L) ^§^	0.15	−
TG (mg/dL) ^§^	<0.01	0.34
Alb (g/dL)	0.67	−
Glu (mg/dL) ^§^	0.11	−
Insulin (μU/mL) ^§^	<0.01 *	0.27
HbA1c (%)	0.12	−
AST (U/L)	0.51	−
ALT (U/L) ^§^	0.02 *	0.21

This table shows the correlation between estimated salt intake and sarcopenia-related parameters. As for the correlation coefficient, the Pearson correlation coefficient was shown in the case of normality, and the Spearman correlation coefficient was shown in the case of non-normality [§]. *: *p* < 0.05. sec: seconds, BW: body weight, SBP: systolic blood pressure, DBP: diastolic blood pressure, BMI: body mass index, SMI: skeletal muscle mass index, ULMM: mass of muscle of upper limbs, LLMM: mass of muscle of lower limbs, BFP: body fat percentage, HGS/BW: handgrip strength/weight, KES: knee extensor muscle strength, SLT: single-leg stance time, MGS: maximum gait speed, Flex: long seat type body anteflexion, 30CS: chair rise test, IL-6: Interleukin-6, UN: urea nitrogen, CysC: cystatin C, TG: triglyceride, Alb: albumin, Glu: glucose, HbA1c: Glycosylated hemoglobin, AST: Aspartate transaminase, ALT: Alanine aminotransferase.

**Table 3:** Comparison of sarcopenia-related parameters by estimated salt intake and age.

	Younger Low—Salt	Younger High—Salt	Older Low—Salt	Older High—Salt	*p* value
Sample size (*n*)	26	25	31	32	–
Salt intake (g/day)	7.56(1.19)	11.01(1.08)	7.67(1.42)	11.19(1.23)	–
Male/Female (*n*)	5/21	6/19	6/25	2/30	–
Age (year) ^§^	48.50[37.75–53.00]	50.00[44.50–54.00]	62.00[58.00–71.00]	65.00[60.25–67.00]	–
SBP (mmHg) ^§^	115.33[103.33–122.67]	112.67[103.83–119.83]	112.33[105.75–121.00]	122.50[113.67–130.75]	<0.01 *
DBP (mmHg) ^§^	68.17[60.50–73.75]	68.33[64.17–72.50]	65.33[59.67–71.67]	72.67[67.83–81.50]	<0.01 *
Weight (kg) ^§^	52.50[46.23–62.33]	61.50[52.80–69.00]	47.00[44.80–56.80]	51.15[47.83–62.13]	<0.01 *
BMI (kg/m^2^) ^§^	20.61[19.00–22.50]	23.10[20.50–25.86]	20.45[18.27–21.83]	22.20[20.00–25.61]	<0.01 *
SMI (kg/m^2^) ^§^	6.25[5.78–7.10]	6.40[6.05–7.60]	6.00[5.40–6.70]	5.95[5.63–6.78]	0.01 *
ULMM (kg) ^§^	3.81[3.19–4.71]	3.82[3.56–5.55]	3.28[2.86–4.04]	3.61[2.90–4.13]	0.03 *
LLMM (kg) ^§^	12.44[10.21–14.43]	12.27[11.67–17.10]	11.04[9.92–13.63]	10.86[9.91–12.15]	<0.01 *
BFP (%)	22.54(7.31)	28.38(7.98)	23.76(7.08)	31.12(9.07)	<0.01 *
HGS/BW (kg/BW)	0.56(0.10)	0.49(0.11)	0.52(0.10)	0.44(0.13)	<0.01 *
KES/BW (kg/BW)	0.64(0.20)	0.62(0.16)	0.60(0.14)	0.53(0.16)	0.08
SLT (sec) ^§^	60.00[60.00–60.00]	60.00[48.35–60.00]	60.00[25.12–60.00]	60.00[37.51–60.00]	0.23
MGS (m/sec) ^§^	2.34[2.13–2.79]	2.25[2.05–2.73]	2.24[1.95–2.59]	2.13[1.81–2.33]	0.05
Flex (m)	0.39(0.10)	0.36(0.09)	0.38(0.11)	0.35(0.07)	0.44
30CS (time) ^§^	24.00[19.75–29.00]	20.00[16.00–23.00]	21.00[18.00–29.00]	17.50[16.00–22.00]	<0.01 *
Renalase (mg/L) ^§^	3.71[2.78–6.30]	4.85[2.71–6.28]	4.40[3.37–5.12]	4.13[3.45–4.95]	0.73
IL-6 (pg/mL) ^§^	0.80[0.60–1.03]	0.90[0.75–1.45]	1.00[0.70–1.30]	1.70[1.10–2.08]	<0.01 *
UN (mg/dL)	13.23(2.87)	12.54(2.29)	14.72(2.62)	13.49(2.79)	0.02 *
CysC (mg/L) ^§^	0.63[0.61–0.68]	0.66[0.61–0.69]	0.65[0.62–0.70]	0.68[0.61–0.70]	0.43
TG (mg/dL) ^§^	65.50[46.50–81.75]	84.00[52.50–95.00]	58.00[43.00–78.00]	82.50[66.25–105.75]	0.01 *
Alb (g/dL)	4.50(0.29)	4.54(0.24)	4.55(0.32)	4.49(0.27)	0.74
Glu (mg/dL) ^§^	93.00[87.50–99.25]	97.00[90.50–100.50]	99.00[94.00–108.00]	100.00[94.25–107.75]	0.02 *
insulin (μU/mL) ^§^	4.05[2.98–5.95]	4.90[3.55–6.55]	3.70[2.70–6.80]	5.00[3.98–7.45]	0.10
HbA1c (%)	5.48(0.29)	5.55(0.31)	5.73(0.32)	5.79(0.39)	<0.01 *
AST (U/L)	21.19(4.50)	23.36(5.06)	24.39(4.87)	24.69(5.01)	0.04 *
ALT (U/L) ^§^	16.00[12.00–17.25]	19.00[15.00–29.50]	18.00[15.00–21.00]	16.00[15.00–26.75]	0.05

These data are shown as mean (standard deviation) or median [interquartile range]. This is the result of one-way ANOVA or Kruskal-Wallis tests [§]. *: *p* < 0.05. sec: seconds, BW: body weight, SBP: systolic blood pressure, DBP: diastolic blood pressure, BMI: body mass index, SMI: skeletal muscle mass index, ULMM: mass of muscle of upper limbs, LLMM: mass of muscle of lower limbs, BFP: body fat percentage, HGS/BW: handgrip strength/weight, KES: knee extensor muscle strength, SLT: single-leg stance time, MGS: maximum gait speed, Flex: long seat type body anteflexion, 30CS: chair rise test, IL-6: Interleukin-6, UN: urea nitrogen, CysC: cystatin C, TG: triglyceride, Alb: albumin, Glu: glucose, HbA1c: Glycosylated hemoglobin, AST: Aspartate transaminase, ALT: Alanine aminotransferase.
